# First record of the subfamily Trichomyiinae Tonnoir, 1922 and genus *Trichomyia* Haliday in Curtis, 1839 (Diptera, Psychodidae) in China, with the description of a new extant species

**DOI:** 10.3897/zookeys.1278.183769

**Published:** 2026-04-30

**Authors:** Shuai-Lai Yang, Yi-Da Guo, Zhen Wang, Xin-Ming Yin, Yu-Qiang Xi

**Affiliations:** 1 State Key Laboratory of Wheat and Maize Crop Science, Henan International Laboratory for Green Pest Control, Henan Agricultural University, No. 95 Wenhua Road, Jinshui District, Zhengzhou 450003, Henan Province, China Department of Entomology, Henan Agricultural University Zhengzhou China; 2 Department of Entomology, Henan Agricultural University, No. 95 Wenhua Road, Jinshui District, Zhengzhou 450003, Henan Province, China State Key Laboratory of Wheat and Maize Crop Science, Henan International Laboratory for Green Pest Control, Henan Agricultural University Zhengzhou China

**Keywords:** Chinese, identification key, moth fly, new record, taxonomy

## Abstract

The subfamily Trichomyiinae Tonnoir, 1922 and the genus *Trichomyia* Haliday in Curtis, 1839 are recorded for the first time in China. A new species, *Trichomyia sinensis***sp. nov**., is described, and photographs of an in situ adult male and its habitat are provided. An updated key to *Trichomyia* species from the Palearctic and Oriental regions is presented.

## Introduction

Species of Psychodidae are diverse and abundant, with approximately 3400 known worldwide (Galati and Rodrigues 2023). Psychodidae includes six extant subfamilies: Bruchomyiinae Alexander, 1921; Horaiellinae Enderlein, 1937; Phlebotominae Rondani, 1840; Psychodinae Newman, 1834; Sycoracinae Jung, 1954, and Trichomyiinae Tonnoir, 1922 (Kvifte and Wagner 2017). There are over a thousand species in Psychodinae and Phlebotominae, while the number of species is fewer in the other subfamilies (Galati and Rodrigues 2023; Galati et al. 2025). Trichomyiinae is a subfamily of short-legged psychodids, with the eye bridge absent, antenna with 13 or 14 flagellomeres, the palpus 3- or 4-segmented (the basal ones sometimes fused together), the wing oval, with a single longitudinal vein between the branches R_2+3_ and M_1+2_, and males with terminalia rotated by 180° due to torsion of abdominal segments 7 and 8, which are complex and diverse in shape; females have one pair of spermathecae (Morelli 2025).

There are two extant genera of Trichomyiinae, 199 extant species, and 25 fossil species (Araújo et al. 2017, 2023). The classification system of the extant species of Trichomyiinae has long been contentious. Historically, Duckhouse (1965) informally divided *Trichomyia* into two groups, “Group A” and “Group B”, based on the number of palpus segments and location of sensory pits, and it was recognised for decades as comprising only a single genus. Araújo et al. (2023) proposed a phylogeny of *Trichomyia* based on the morphological characteristics of males, and one of the subgenera, *Gondwanotrichomyia* Duckhouse, 1980, was separated and recognised as an independent genus. However, *Trichomyia* Haliday in Curtis, 1839 remains the most species-rich genus within the subfamily Trichomyiinae, encompassing 188 extant species. *Trichomyia* is characterised by 1) a short distance between antennal sockets, with the width less than one-third of the antennal socket; 2) dorsal border of head bounded by the postoccipital margin and oval or subtriangular opistosomal suture; 3) palpus with four or three segments; when palpus with four segments, the first and second segments fused along a small section of the proximal area of first segment; 4) antennae with the first flagellomere approximately the same length as the second flagellomere; all flagellomeres connected asymmetrically in most species; 5) ascoids long, approximately the same length as the flagellomere or longer than the flagellomere, and located near the flagellomere base; and 6) scutum with alveoli concentrated on the margins (Araújo et al. 2023).

*Trichomyia* currently includes seven subgenera, three of which were described from Australia and New Guinea (*Apotrichomyia* Duckhouse, 1978; *Dactylotrichomyia* Duckhouse, 1978; and *Dicrotrichomyia* Duckhouse, 1978), and four subgenera from the Neotropical region (*Septemtrichomyia* Bravo, 1999; *Opisthotrichomyia* Bravo, 2001; *Syntrichomyia* Araújo & Bravo, 2013; *Brachiotrichomyia* Bravo & Araújo, 2013; see review by Araújo and Bravo 2016). The Afrotropical, Oriental, and Holarctic species have not yet been assigned to subgenera. *Trichomyia* is distributed worldwide, but over half of the species are found in the Neotropical region. Ten species are distributed in the Palaearctic region: *T. carlestolrai* Wagner, 2001, *T. hardeggensis* Omelková & Ježek, 2012, *T. itocoae* Tokunaga & Komyo, 1956, *T. kostovi* Ježek, 1990, *T. kviftei* Morelli, 2018, *T. malickyi* Wagner, 1982, *T. minima* Withers, 2004, *T. parvula* Szabó, 1960, *T. stephani* Beran, Doczkal, Pfister & Wagner, 2010, and *T. urbica* Haliday in Curtis, 1839; six species are distributed in the Oriental region: *T. batu* Quate, 1962, *T. caelibata* Quate, 1965, *T. malaya* Quate, 1962, *T. ransangi* Quate, 1965, *T. trifida* Quate, 1965, and *T. triflis* Quate, 1965 (Quate 1962, 1965; Wagner 1982, 2001; Ježek 1990; Withers 2004; Beran et al. 2010; Omelková and Ježek 2012; Morelli 2018, 2025) Before this study, only one fossil species, *T. duckhousei* Wang, Zhang & Azar, 2011, was recorded from China, and no extant species of Trichomyiinae had been reported in China (Wang et al. 2011).

In this paper, we describe and illustrate the new species, *T. sinensis* sp. nov., which represents the first discovery of an extant species from the Trichomyiinae and the genus *Trichomyia* in China. We provide photographs of in situ adults and habitat from the type locality, along with an identification key to the species of *Trichomyia* in the Palaearctic and Oriental regions. Notably, this species does not belong to any currently recognised subgenus, and its taxonomic position within the genus requires further exploration.

## Materials and methods

The head, wings, and male genitalia were separately dissected. Genitalia preparations were made by removing and macerating the apical portion of the abdomen in glacial acetic acid, rinsing in distilled water, and stored in glycerine-filled microvials. After examination, the head, wings and genitalia were all preserved in small tubes filled with glycerol.

Specimens were examined and photographed using a Leica M205A stereomicroscope. Helicon Focus v. 7.0.2 was used for image stacking. Scale drawings were created based on the photographs, with partial structural details observed and refined under a stereomicroscope through adjustments and modifications before finalization. In situ photographs of the adult were taken with Sony a7m3 and Sony 90 mm macro lens, while environmental photos of the collection site were captured using a mobile phone (Redmi K60). Image plates were post-processed with Adobe Photoshop CC 2019 Extended.

All measurements of adults are in millimetres (mm). The palpus formula was calculated with the first segment as unit 1.

Specimens examined were deposited in the Entomological Museum of Henan Agricultural University (**HAU**), Zhengzhou. General morphological terminology follows that of Cumming and Wood (2017). Terminology for the wing follows Wagner and Ibáñez-Bernal (2009). Male terminalia follows Araújo and Bravo (2016), and we use their term “aedeagal projections” to describe the complex structure within the male aedeagal complex. Following Morelli (2018), we use their term “ parameral sheath” to describe the sclerotised plate that connected to gonocoxites in ventral view, medially fused and antero-laterally extended, forming a U-shaped structure.

## Taxonomy

### Key to the species of *Trichomyia* of Palaearctic and Oriental regions

**Table:** 

1	More or less reduced articulation between second and third flagellomere of antenna; dorsal arm of gonocoxite digitiform; gonocoxal apodemes with a pair of subcircular projections in distal region, close to gonocoxite	**2 (subgenus *Dactylotrichomyia* Duckhouse)**
–	Articulation between second and third flagellomere of antenna not reduced; gonocoxite not digitiform; gonocoxal apodemes without a pair of subcircular projections	**4**
2	Aedeagus furcate, with subapical spurs	***T. malaya* Quate, 1962 (Malaysia)**
–	Aedeagus unbranched, with spurs	**3**
3	R-fork on same level as CuA_1_ apex; gonocoxite with 3 distal processes	***T. trifida* Quate, 1965 (Philippines)**
–	R-fork basad of CuA_1_ apex; gonocoxite with 2 distal processes	***T. triflis* Quate, 1965 (Philippines)**
4	Palpus with 4 segments; first and second segments fused or not fused	**5**
–	Palpus with 3 segments	**9**
5	Palpus with first and second segments fused	**6**
–	Palpus with first and second segments separate	**8**
6	Last segment of palpus longer than first and second segments combined; apex of wing pointed, R-fork close to base of wing	***T. batu* Quate, 1962 female (Malaysia)**
–	Last segment of palpus shorter than sum of first and second segments; apex of wing wide and round, R-fork close to margin of wing	**7**
7	Vein r-m absent; gonostylus cylindrical, distally not bifurcated	***T. kviftei* Morelli, 2018 (Italy)**
–	Vein r-m present; gonostylus distally bifurcated	***T. sinensis* sp. nov**.
8	Vein Sc curved towards costal margin, vein r-m absent; gonostylus straight, distally with short setae	***T. carlestolrai* Wagner, 2001 (Spain)**
–	Vein Sc straight, vein r-m present; gonostylus curve, distally without setae	***T. urbica* Haliday in Curtis,1839 (widely distributed in Europe)**
9	First segment of flagellomeres elongate, at least twice as long as other segments; gonostylus with many short, strong spines on inner side	***T. parvula* Szabó, 1960 (Czech Republic, France, Hungary, Germany, Great Britain, and Italy)**
–	Flagellomeres subequal in length, with longest first flagellomere not exceeding 1.5× length of others; gonostylus spineless or sparsely spined	**10**
10	First segment of the palpus twice as long as second segment; gonostylus with setae only at its apex, with remaining portion glabrous, slender, and exceeding length of gonocoxite	***T. minima* Withers, 2004 (England)**
–	First segment of the palpus not reaching twice length of second segment (1.3–1.9×); gonostylus either not exceeding length of gonocoxite or entirely densely setose	**11**
11	Vein sc-r absent	**12**
–	Vein sc-r present	**13**
12	Vein Sc curved towards costal margin; gonostylus bifurcate, longer part pointed to form a very sclerotised bent hook apically, shorter part with a rather blunt top	***T. kostovi* Ježek, 1990 (Bulgaria and European Russia)**
–	Vein Sc straight; gonostylus cylindrical, with long setae, slightly medially bent	***T. malickyi* Wagner, 1982 (Greece)**
13	R_4+5_ complete, vein r-m present	**14**
–	R_4+5_ incomplete, vein r-m absent	**15**
14	First segment of palpus without circular sensory depression; cercus stout, upcurved, setigerous entirely, with chitinised ridge on concave side of each cerci, apical setae	***T. itocoae* Tokunaga & Komyo, 1956 (Japan)**
–	First segment of palpus with circular sensory depression; cercus elongate, triangular, quite large and setose, best visible in lateral view	***T. stephani* Beran, Doczkal, Pfister & Wagner, 2010 (Germany)**
15.	Gonocoxites swollen, distally with 2 processes: a long, prominent ventral process, covered with strong bristles along its whole inner margin, and a not so prominent short, broad ventral process with several tips bearing strong setae	***T. hardeggensis* Omelková & Ježek, 2012 (Czech Republic)**
–	Gonocoxite composed of 2 processes approximately similar in shape	**16**
16	Base of R_4+5_ and M_3_ nearly on same level; gonocoxites composed of 2 processes, with upper a little longer and more slender than lower	***T. ransangi* Quate, 1965 (Philippines)**
–	Base of R_4+5_ distinctly distad of M_3_ base; gonocoxites composed of 2 processes approximately similar in shaped, with upper acutely pointed and lower blunt	***T. caelibata* Quate, 1965 (Philippines)**

#### Trichomyia sinensis sp. nov.

Taxon classificationAnimaliaDipteraPsychodidae

2B9168F2-1BEB-5A9B-B0E6-681D10F5CB37

https://zoobank.org/DB1AE641-E3A3-4F5A-B05B-F75058D02AFB

**Figure 1.**
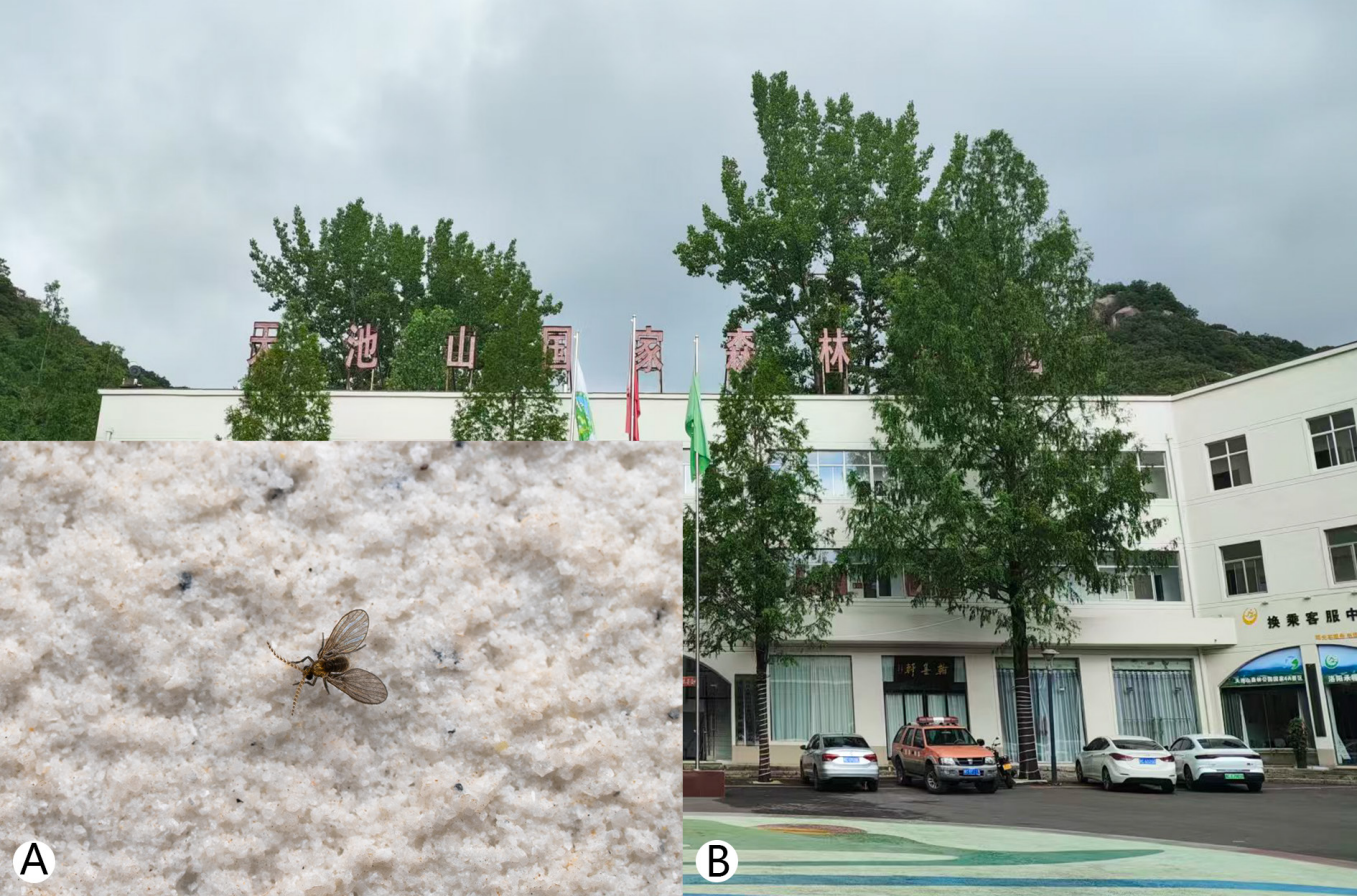
*Trichomyia sinensis* sp. nov. and habitat. **A**. *T. sinensis* in situ; **B**. Tourist Reception Centre, Tianchishan National Forest Park, Song County, Luoyang City, Henan Province, China.

**Figure 2.**
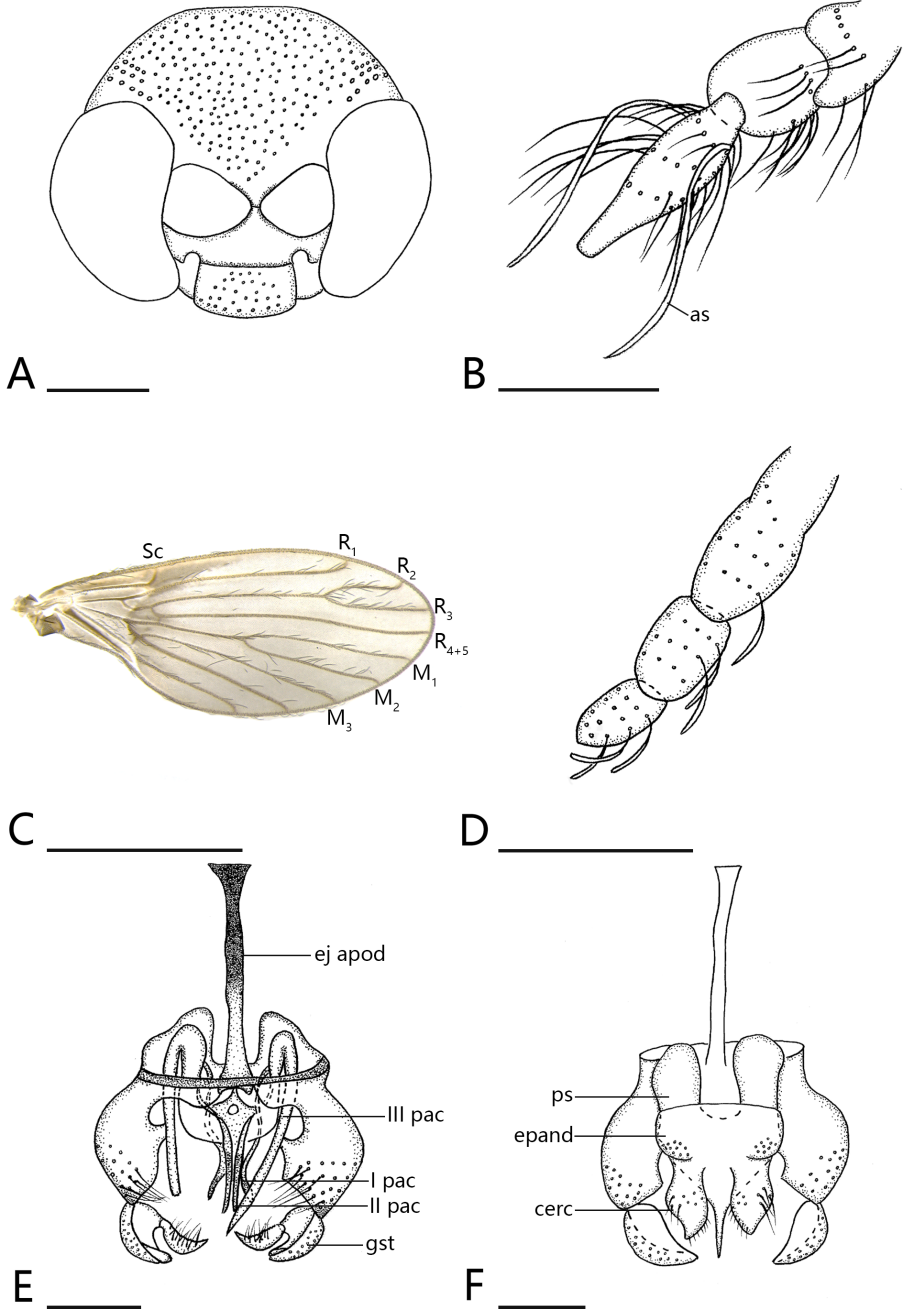
*Trichomyia sinensis* sp. nov. **A**. Head, lateral view; **B**. Antennae; **C**. Wing; **D**. Palpus, lateral view; **E**. Aedeagal complex, dorsal view; **F**. Aedeagal complex, ventral view. Scale bars: 1 mm (**C**); 0.1 mm (**A, B, D–F**). Abbreviations: as – ascoids; ej apod – ejaculatory apodeme; I pac, II pac, III pac – projections in aedeagal complex I, II and III; gst – gonostylus; ps – parameral sheath; epand – epandrium; cerc- cercus.

**Figure 3.**
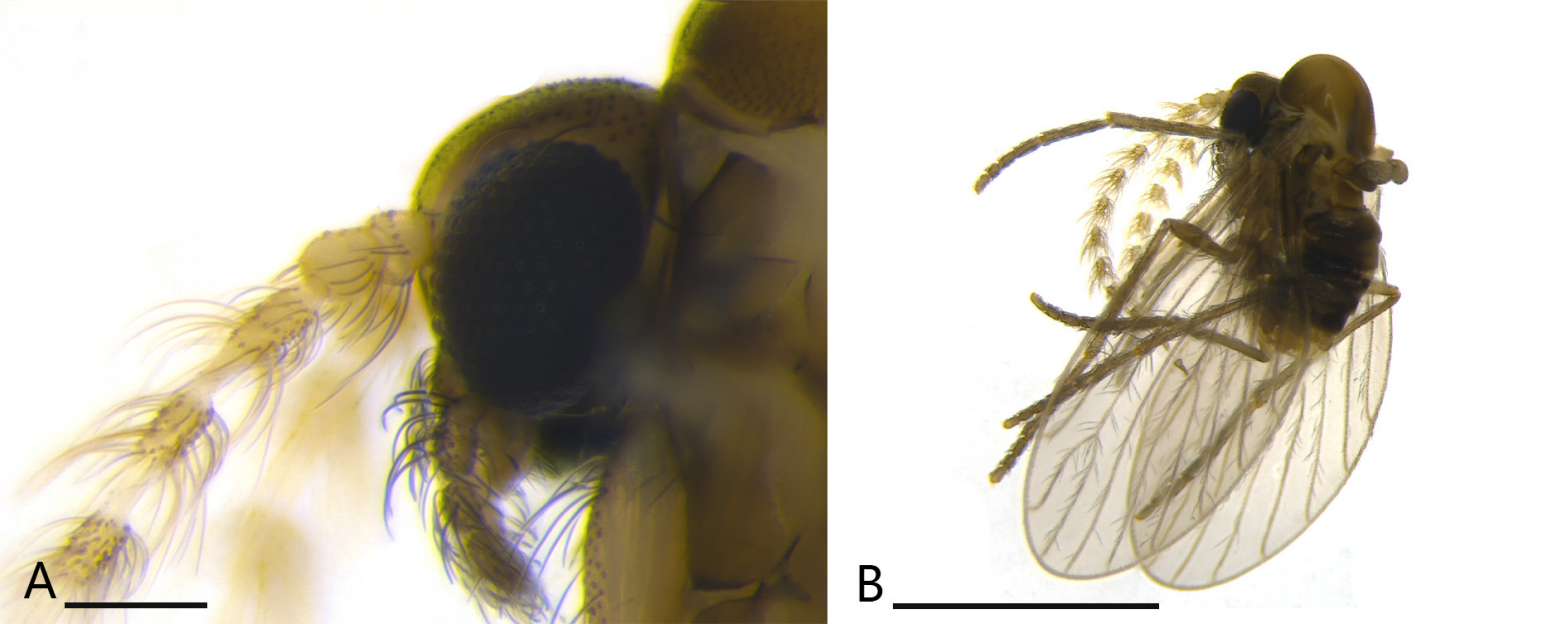
*Trichomyia sinensis* sp. nov., male holotype. **A**. Head lateral view; **B**. Overall lateral view. Scale bars: 1 mm (**B**); 0.1 mm (**A**).

Figs 1A, 1B, 2A–F, 3A, 3B

##### Type material.

***Holotype***: • 1♂, China, Henan Province, Xinxiang City, Hui County, Baligou Scenic Area, Xilian Scenic Area, 35°38'39.33"N, 113°33'24.43"E, 871 m elev., 2025.VIII.16, leg. Shuailai Yang. ***Paratypes***: • 1♂, China, Henan Province, Xinxiang City, Hui County, Baligou Scenic Area, 35°35'26.2"N, 113°32'14.44"E, 717 m elev., 2025.VIII.15, leg. Kailin Wang; • 1♂, China, Henan Province, Luoyang City, Song County, Tianchishan National Forest Park, Tourist Reception Center, 34°15'36.89"N, 111°50'58.74"E, 1020 m elev., 2025.VI.29, leg. Shuailai Yang.

##### Diagnosis.

Palpus with four segments; first and second segments fused at articulation, without sensilla. Sc, R_1_, and R_2_ apically curved towards costal margin; radial fork positioned closer to wing margin than median fork; r-m and sc-r present. Gonostylus incurved; dorsal surface pilose; ventral surface glabrous; apex bifurcated; one ramus falcate and marginally serrulate, bearing elongate setae along inner margin. Aedeagal complex with two pairs of sclerotised projections: one pair small and pointed, other pair broad bent at apex, slender, pointed, extending towards end of aedeagal complex.

##### Description.

Measurements (*N* = 1). **Male**. Body length 1.09 mm. Wing length 1.97 mm, width 0.809 mm.

***Head*** (Figs 2A, 3A) suboval in frontal view, width 0.392 mm, length 0.287 mm; vertex 0.098 mm; eyes without eyebridge. Supraocular bristles arranged in multiple rows. Antennal sockets subtriangular. Antennae (Fig. 2B) in type specimens all incomplete, with longest fragment consisting of 11 segments; scape length 0.05 mm; pedicel subcircular length 0.07 mm, width 0.054 mm; flagellomeres fusiform, swollen at middle and distally narrowing. Flagellomeres 1–9: 0.12: 0.119: 0.121: 0.111: 0.117. 0.114: 0.107: 0.118: 0.117. Ascoids transparent, long, slender, sharply pointed apex, 1.05 times length of flagellomeres. Palpus (Fig. 2D) with four segments: first and second segments fused at articulation, without sensilla; palpus formula 1.0: 1.87: 1.78: 2.3.

***Thorax*** mesonotum without setae in region of median line, dark brown along both sides of median line, densely setose; remaining mesonotum light brown. Scutellum with lateral setae clustered near apical angles, with a single row of lateral setae along margins, otherwise completely without setae. Pleuron surface entirely surface. Wing (Fig. 2C) brownish; veins Sc, R_1_, and R_2_ apically curved towards costal margin; vein sc-r short and inconspicuous; radial and medial forks complete; radial fork positioned closer to wing margin than median fork; r-m present. Vein R_4+5_ complete at base, slightly below top the wing.

***Terminalia*** (Fig. 2E, F).

Yellow, except for black upper half of ejaculatory apodeme. Hypandrium and gonocoxites separate; hypandrium narrow; gonocoxite length 0.1 mm, slightly wider and rounder at middle, with multiple, long setae at end. Gonostylus incurved, with dorsal surface pilose and ventral surface glabrous, apex bifurcated, one ramus falcate and marginally serrulate, bearing elongate setae along inner margin. Aedeagal complex with three pairs of sclerotised projections: first pair near sides of aedeagus, small, and pointed; second pair slightly forked, sharp, and at end of aedeagus; third pair broad and bent at apex, extending towards end of slender, pointed aedeagal complex and 0.24 mm long, significantly longer than aedeagus. Ejaculatory apodeme 0.26 mm long, 1.6× longer than aedeagus, rod-shaped, long, thin, broad at apex in lateral view. Aedeagus slender, slightly separate, and distally pointed. Parameral sheath large in ventral view, completely smooth, apically broad, with distal end gradually narrower and more pointed. Epandrium approximately rectangular, width 1.78× times length. Hypoproct absent. Cercus pyriform, separate; outer surface densely setose; inner apical margin with small setae.

**Female**. Unknown.

##### Distribution.

China (Henan).

##### Etymology.

The specific name refers to China, as the new species was first discovered there.

##### Biology.

**Figure 4.**
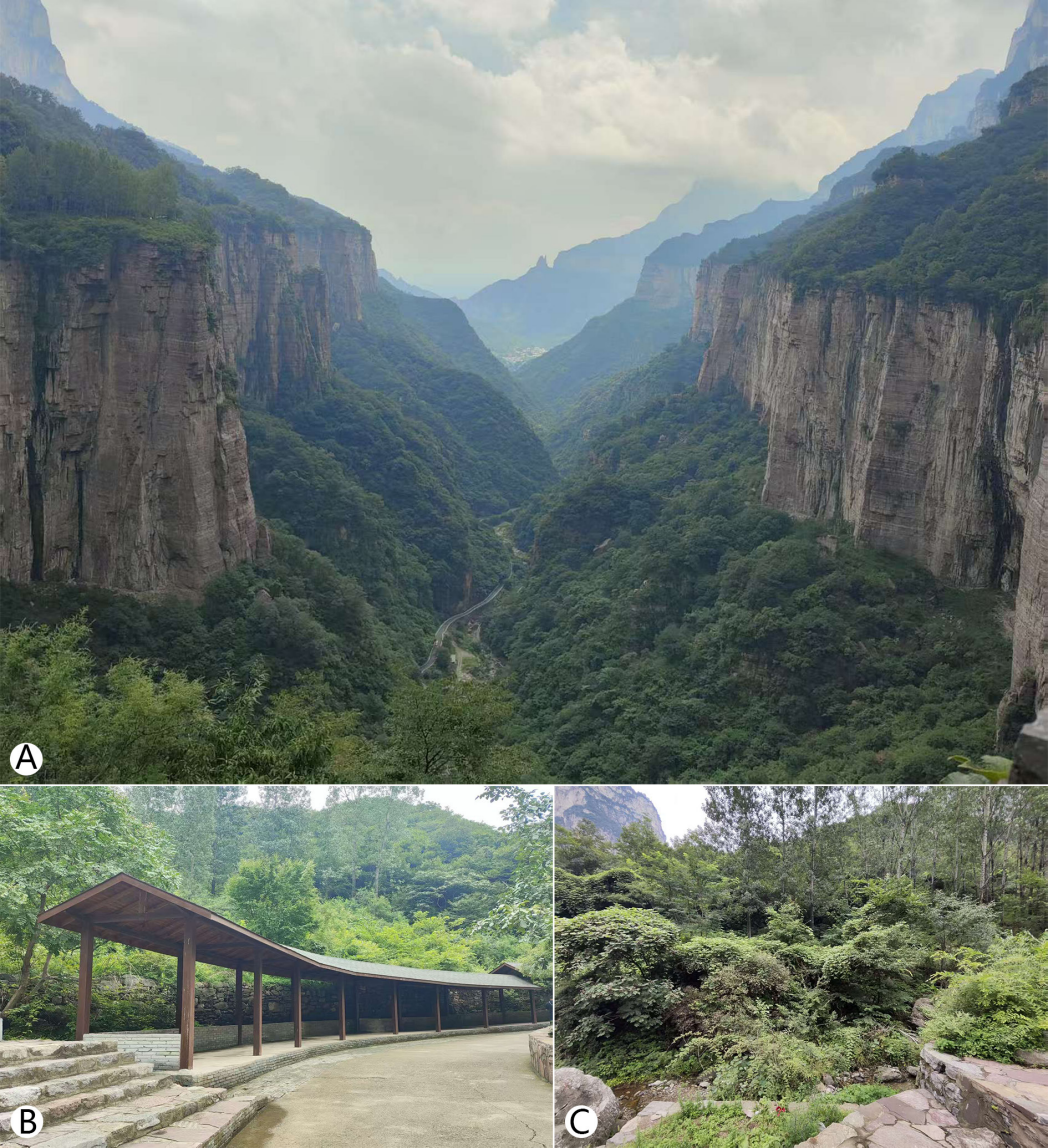
*Trichomyia sinensis* sp. nov. specimen collection site in Xilian Scenic Area, Baligou Scenic Area, Henan Province, China. **A**. Valley at collection site; **B**. Pavilion at collection site; **C**. Surrounding habitat.

**Figure 5.**
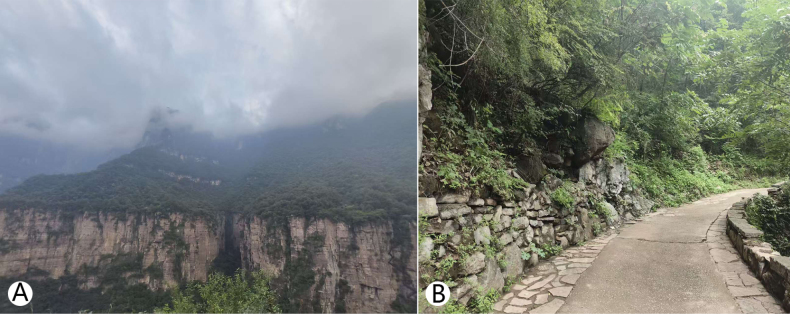
*Trichomyia sinensis* sp. nov. specimen collection site in Xilian Scenic Area, Baligou Scenic Area, Henan Province, China. **A**. Collection area, mountain range; **B**. Trails and vegetation at collection site.

The specimen from the Tianchishan National Forest Park was found at night on the walls of the Tourist Reception Centre (Fig. 1B), where surrounding house lights likely attracted them. The other two specimens were collected using a sweep net during overcast, rainy weather in humid environments (Figs 4, 5). The specimen from Xilian Scenic Area was obtained by sweep netting in the gap between a pavilion and its back wall, an area containing abundant humus and some garbage, and with a stream nearby (Fig. 4B, C).

##### Remarks.

The new species resembles *T. urbica* in the habitus, incurved gonostylus, bifurcated apex, and rod-shaped ejaculatory apodeme, but it can be separated from that species by the following: palpus with four segments, first and second segments fused at the articulation; wing r-m and sc-r present; and gonostylus with one of the rami falcate and marginally serrulate, bearing elongate setae along the inner margin. In *T. urbica*, the palpus has four segments, but the first and second segments are separated, the r-m vein is absent, and the gonostylus laterally has a long, sharp edge, which ends in a hook; the distal part is medially bent, ending in a rounded tip (Wagner 1982).

## Discussion

The genus *Trichomyia* currently is divided into several subgenera. Three species from the Oriental region—*T. malaya*, *T. trifida*, and *T. triflis*—are assigned to the subgenus *Dactylotrichomyia*, but the remaining species from the Palearctic and Oriental regions have not yet been definitively assigned to subgenera (Araújo et al. 2023). The morphological characters of *Trichomyia sinensis* sp. nov. do not align with any existing subgenus. Notably, certain characteristics of the palpus, gonostylus, and aedeagal complex do not match the diagnoses of all existing subgenera and are unique to this new species. These differences may represent common diagnostic features of a new subgenus.

The discovery of *Trichomyia sinensis* sp. nov. in Henan Province, in North China and straddling the boundary between northern and southern China, underscores the importance of exploring understudied regions. Numerous potential new species may exist across China, which could significantly enhance our understanding of the genus *Trichomyia* and the subfamily Trichomyiinae.

The fossil *T. duckhousei* was discovered by Wang et al. (2011) and represents the first fossil record of *Trichomyia* in China. Abundant fossil evidence indicates that this taxonomic group had already developed considerable diversity by Cretaceous. Wang et al. (2011) also hypothesised that extant species of Trichomyiinae likely exhibit significant richness. The discovery of extant Trichomyiinae species in China conclusively demonstrates both the remarkable diversity of this taxonomic group within the region and the substantial unexplored potential for identifying new species. This discovery has also significantly filled the gaps in our understanding of the biogeography of the Trichomyiinae and crucially could provide a basis for a more complete knowledge of their phylogeny.

## Supplementary Material

XML Treatment for Trichomyia sinensis

## Additional information

### Artificial Intelligence (AI) use

The authors accept full responsibility for the content of the manuscript, including the disclosure of any use of AI.

No AI tools were used in the preparation of this manuscript.

### Author ORCIDs

S.-L. Yang https://orcid.org/0009-0002-6813-8949

Y.-D. Guo https://orcid.org/0009-0002-7863-7469

Z. Wang https://orcid.org/0009-0009-3808-5429

X.-M. Yin https://orcid.org/0000-0003-3936-3557

Y.-Q. Xi https://orcid.org/0000-0002-3285-8904

### Data availability

All of the data that support the findings of this study are available in the main text.
